# Nutritional Value, Chemical Composition and Cytotoxic Properties of Common Purslane (*Portulaca oleracea* L.) in Relation to Harvesting Stage and Plant Part

**DOI:** 10.3390/antiox8080293

**Published:** 2019-08-08

**Authors:** Spyridon A. Petropoulos, Ângela Fernandes, Maria Inês Dias, Ioannis B. Vasilakoglou, Konstantinos Petrotos, Lillian Barros, Isabel C. F. R. Ferreira

**Affiliations:** 1 Department of Agriculture Crop Production and Rural Environment, University of Thessaly, 38446 N. Ionia, Magnissia, Greece; 2 Centro de Investigação de Montanha (CIMO), Instituto Politécnico de Bragança, Campus de Santa Apolónia, 5300-253 Bragança, Portugal; 3 Department of Crop Production-Agrotechnology, University of Thessaly, 41110 Larissa, Greece

**Keywords:** α-linolenic acid, fatty acids, hepatotoxicity, omega-3 fatty acids, *Portulaca oleraceae*, phenolic compounds, oleracein derivatives, purslane, tocopherols

## Abstract

Purslane (*Portulaca oleraceae* L.) is a widespread weed, which is highly appreciated for its high nutritional value with particular reference to the content in omega-3 fatty acids. In the present study, the nutritional value and chemical composition of purslane plants in relation to plant part and harvesting stage were evaluated. Plants were harvested at three growth stages (29, 43 and 52 days after sowing (DAS)), while the edible aerial parts were separated into stems and leaves. Leaves contained higher amounts of macronutrients than stems, especially at 52 DAS. α-tocopherol was the main isoform, which increased at 52 DAS, as well total tocopherols (values were in the ranges of 197–327 μg/100 g fresh weight (fw) and 302–481 μg/100 g fw, for α-tocopherol and total tocopherols, respectively). Glucose and fructose were the main free sugars in stems and leaves, respectively, whereas stems contained higher amounts of total sugars (values were ranged between 0.83 g and 1.28 g/100 g fw). Oxalic and total organic acid content was higher in leaves, especially at the last harvesting stage (52 DAS; 8.6 g and 30.3 g/100 g fw for oxalic acid and total organic acids, respectively). Regarding the fatty acid content, stems contained mainly palmitic (20.2–21.8%) and linoleic acid (23.02–27.11%), while leaves were abundant in α-linolenic acid (35.4–54.92%). Oleracein A and C were the major oleracein derivatives in leaves, regardless of the harvesting stage (values were in the ranges of 8.2–103.0 mg and 21.2–143 mg/100 g dried weight (dw) for oleraceins A and C, respectively). Cytotoxicity assays showed no hepatotoxicity, with GI_50_ values being higher than 400 μg/mL for all the harvesting stages and plant parts. In conclusion, early harvesting and the separation of plant parts could increase the nutritional value of the final product through increasing the content of valuable compounds, such as omega-3 fatty acids, phenolic compounds and oleracein derivatives, while at the same time, the contents of anti-nutritional compounds such as oxalic acid are reduced.

## 1. Introduction

Purslane (*Portulaca oleracea* L.) is a widespread weed belonging to the Portulacaceae family, with extensive distribution throughout the world [1]. It is also a basic component of the so-called Mediterranean diet and an ingredient of many salad dishes [2]. Although it is considered a very invasive weed [3], it is also highly appreciated for the high nutritional value of its edible plant parts, due to their high content in omega-3 fatty acids and, particularly, in α-linolenic acid [4]. Other valuable components of purslane edible parts include minerals, such as calcium, potassium and phosphorus, macronutrients such as proteins and carbohydrates [5], as well as tocopherols, carotenoids and ascorbic acid [6,7]. Moreover, phenolic compounds and oleracein derivatives of extracts of purslane leaves have been attributed with antioxidant properties [8], while their content may be affected by cooking and food additives [9]. *P. oleracea* is tolerant under stress conditions, such as heat, drought and salinity stress, a trait that could be useful within the ongoing climate change context and provide alternative solutions to farmers in climate-affected regions [10]. 

Several studies have reported the effects of cultivation practices, growing conditions and genetic factors on the chemical composition of purslane. In particular, Rahdari et al. [11], Uddin et al. [12] and Teixeira and Carvalho [13] reported that salinity stress may significantly affect proximate and mineral composition, while salinity stress also has an impact on polyphenol and carotenoid content and antioxidant activity [14]. Genetic material is also important for obtaining edible parts of high nutritional value, since many studies have suggested significant differences in the chemical compositions of purslane accessions and genotypes, especially regarding fatty acid and oxalic acid content [6,15] and bioactive properties [16]. Cultivation practices such as planting date or fertilization rates can also be proven as useful means towards the modulation of the chemical composition of purslane edible parts [17,18]. In addition, cultivation systems may affect the nutritional value of purslane aerial parts, with soilless culture showing promising results in regards to improving fatty acid composition and decreasing oxalic acid content [19]. High oxalic acid content is considered as an antinutrient factor, and apart from cultivation systems, the proper harvesting stage and cultivar selection, as well as the modulation of nutrient solution composition may also be helpful in decreasing its content and increasing the overall nutritional value of purslane edible parts [15,20]. Harvesting stage and plant parts also have a significant impact on macronutrients (total solids, proteins, ash, and carbohydrates) and mineral content [5], as well as on phenolic compounds, oleracein derivatives and organic acid profile [4]. 

The aim of the present work was to study the effect of harvesting stage on the chemical composition of purslane edible parts, as well as the distribution of the main nutrient components and phytochemicals within the aerial parts of the plant (leaves and stems). Considering the importance of omega-3 fatty acids and oxalic acid for purslane’s nutritional value, special attention was given to oxalic acid and fatty acid content, along with the plant part selection and harvesting stage, which could be used as simple cultivation practices to increase the nutritional and added value of the final product. Finally, the characterization of phenolic compounds and oleracein derivatives in the aerial plant parts (leaves and stems) in relation to harvesting stage was also performed. 

## 2. Materials and Methods 

### 2.1. Plant Material and Growing Conditions

This trial was carried out at the experimental field of the University of Thessaly, in Larissa (Greece; 39°37’18.6” N, 22°22’55.1” E), during the summer of 2016. Seeds of common purslane (*Portulaca oleracea* L.) were obtained from Hortus Sementi Srl. (Budrio, Italy) and were sown directly in soil on 06/06/2016. Prior to sowing, a base dressing of 100 kg/ha with 10-10-10 fertilizer (N-P-K) was applied. Irrigation was applied with sprinklers at regular intervals (once a week, starting on the day of sowing). The soil was sandy clay loam (38% sand, 36% silt, and 26% clay), with pH = 7.4 (1:1 soil/H20) and organic matter content = 1.3%. No pesticides or other agrochemicals were applied during cultivation. Harvesting took place at three different growth stages, namely on 05/07/2016 (29 days after sowing (DAS)), on 19/07/2016 (43 DAS), and on 28/07/2016 (52 DAS). 

After each harvesting stage, the aerial plant parts were divided in stems and leaves. Fresh samples of plant tissues were placed in a forced-air oven, and dry weight was recorded after drying the samples at 70 °C until constant weight. Batch samples of fresh plant tissues were stored at −80 °C and were then lyophilized. The lyophilized samples were ground to powder with a pestle and mortar, and were put in plastic air-sealed bags and stored at −80 °C until further analysis.

For phenolic and oleracein composition, as also for cytotoxicity, a hydroethanolic extract was prepared using 1 g of dried sample with 30 mL ethanol/water (80:20 *v/v*), under magnetic stirring for 1 h. After filtration through a Whatman filter paper N°. 4, the plant residue was re-extracted and the combined filtrates were evaporated at 40 °C (rotary evaporator Büchi R-210, Flawil, Switzerland) and subsequently lyophilized to obtain a dry extract.

### 2.2. Chemical Analyses 

#### 2.2.1. Standards and Reagents

Acetonitrile 99.9% of HPLC grade was from Fisher Scientific (Lisbon, Portugal). Ellipticine was purchased from Sigma-Aldrich (St. Louis, MO, USA), as also were acetic acid, sulforhodamine B (SRB), trypan blue, trichloroacetic acid (TCA), and Tris-(hydroxymethyl)aminomethan (TRIS). Phenolic compound standards were from Extrasynthèse (Genay, France). RPMI-1640 medium, fetal bovine serum (FBS), Hank’s balanced salt solution (HBSS), l-glutamine, nonessential amino acids solution (2 mM), penicillin/streptomycin solution (100 U/mL and 100 mg/mL, respectively), and trypsin-EDTA (ethylenediaminetetraacetic acid) were from Hyclone (Logan, UT, USA). All other chemicals were of analytical purity and obtained from common suppliers. Water was treated via the purification system Milli-Q water (TGI Pure Water Systems, Greenville, SC, USA). 

#### 2.2.2. Nutritional Compounds and Energetic Value

Nutritional compounds of the samples were analyzed (moisture, fat, ash, proteins and carbohydrates) following the Association of Analytical Communities (AOAC) procedures [21]. Briefly, moisture content was determined using an air oven at 105 ± 5 °C until constant weight. Crude protein was evaluated by the macro-Kjeldahl method (N × 6.25) using an automatic distillation and titration unit (model Pro-Nitro-A, JP Selecta, Barcelona, Spain), ash content was determined by incineration at 600 ± 15 °C, and the crude fat was determined by extraction with petroleum ether using a Soxhlet apparatus (Behr Labor Technik, Dusseldorf, Germany). Total carbohydrates were determined by the difference according to the equation: total carbohydrates (g/100 g fresh weight (fw)) = 100 − (g moisture + g fat + g ash + g proteins).

Energy was determined according to the Atwater system following the equation: (kcal/100 g fw) = 4 × (g proteins + g carbohydrates) + 9 × (g fat).

#### 2.2.3. Tocopherols

Tocopherols were determined in the lyophilized samples following a procedure previously described by Dias et al. [22]. The separation of compounds was performed using a high performance liquid chromatography system (Knauer, Smartline system 1000, Berlin, Germany) coupled to a fluorescence detector (FP-2020; Jasco, Easton, PA, USA) programmed for excitation at 290 nm and emission at 330 nm, using the internal standard (IS; tocol, Matreya, Pleasant Gap, PA, USA) method for quantification. After separation, the compounds were identified by comparison with authentic standards. The quantification was based on the fluorescence signal response of each standard, using the internal standard method. The results were expressed in µg per 100 g of fresh weight (fw).

#### 2.2.4. Free Sugars

The lyophilized samples were extracted using a methodology previously described by the authors [23]. The separation of compounds was performed using a HPLC system (Knauer, Smartline system 1000, Berlin, Germany) coupled with a refraction index detector (Knauer Smartline 2300). The mobile phase consisted of an acetonitrile:water mixture (70:30 *v/v*, acetonitrile HPLC-grade, Lab-Scan, Lisbon, Portugal), and separation was achieved using a Eurospher 100-5 NH2 column (4.6 × 250 mm, 5 µm, Knauer, Berlin, Germany). After separation, the compounds were identified by comparison with standards and quantification was performed by the IS method (melezitose). Results were processed using the Clarity 2.4 software (DataApex, Prague, Czech Republic) and expressed in g per 100 g of fw.

#### 2.2.5. Organic Acids

The lyophilized samples were extracted using a methodology previously described and optimized by the authors [23]. The analysis was performed by Ultra-Fast Liquid Chromatography coupled with a Diode-Array Detector (UFLC-DAD; Shimadzu 20A series UFLC, Shimadzu Corporation, Kyoto, Japan). Compounds were identified and quantified by comparison of the retention time, spectra and peak area, recorded at 215 nm, with those obtained from commercial standards. The results were recorded and processed using LabSolutions Multi LC-PDA software (Shimadzu Corporation, Kyoto, Japan) and were expressed in g/100 g fw. 

#### 2.2.6. Fatty Acids

Fatty acids were determined by gas-liquid chromatography with flame ionization detection (GC-FID; DANI1000, Contone, Switzerland), after the extraction and derivatization procedures previously described by Obodai et al. [24]. Fatty acid identification and quantification were performed by comparing the relative retention times of fatty acid methyl ester (FAME) peaks of the tested samples with commercial standards (fatty acid methyl esters (FAMEs) reference standard mixture 37, Sigma-Aldrich, St. Louis, MO, USA). Results were expressed as the relative percentage for each detected fatty acid, using CSW 1.7 software (Data Apex 1.7, Prague, Czech Republic).

#### 2.2.7. Phenolic Compounds and Oleracein Derivatives

Phenolic compounds and oleracein derivatives were evaluated using an ultra-performance liquid chromatography (UPLC) system equipped with a diode array detector (280 nm, 330 nm and 370 nm as preferred wavelengths) coupled to an electrospray ionization mass spectrometry detector (MS) (Dionex Ultimate 3000 UPLC and Linear Ion Trap LTQ XL, Thermo Scientific, San Jose, CA, USA), operating under the conditions described by Bessada et al. [25]. Data acquisition was carried out with the Xcalibur^®^ data system. The identification was made by comparison of retention times, UV-VIS and mass spectra of the sample compounds with those obtained from the available standards, as also with reported data from the literature, and tentatively identified by using the fragmentation pattern. The estimation of phenolic compounds and oleracein derivatives was carried out using the calibration curves obtained from standards, which were constructed based on their UV-VIS signals; the quantification of all compounds was performed at 330 nm. In the case of a non-available standard compound, the most similar structural compound available in the laboratory was used to perform the quantification. A manual integration using the baseline to valley integration mode with baseline projection was performed to obtain the area of the peaks. The results were expressed in mg/100 g dried weight (dw).

### 2.3. Cytotoxicity

#### 2.3.1. Cytotoxicity in Non-Tumor Liver Cell Primary Culture

Hepatotoxic activity was evaluated following the method described by Abreu et al. [26], using a primary cell culture (PLP2) prepared from a porcine liver. Extracts were tested at a final concentration range from 400 to 1.56 μg/mL. Briefly, a cell culture was prepared from a freshly harvested porcine liver (obtained from a local slaughter house) and designated as PLP2 [26]. Briefly, the liver tissue was rinsed in Hank’s balanced salt solution containing 100 U/mL penicillin + 100 μg/mL streptomycin, and was divided into 1 × 1 mm^3^ explants. Some of them were placed into 25 cm^2^ tissue flasks containing Dulbecco’s Modified Eagle’s medium (DMEM, supplemented with 10% fetal bovine serum, 2 mM non-essential amino acids, 100 U/mL penicillin, and 100 mg/mL streptomycin) and incubated at 37 °C under a humidified atmosphere with 5% CO_2_. A phase contrast microscope was used for direct monitoring of the cell cultivation every 2 to 3 days. Before reaching the confluence, cells were subcultured and plated in 96-well plates at a density of 1.0 × 10^4^ cells/well, and cultivated in commercial DMEM medium supplemented with 10% FBS, 100 U/mL penicillin and 100 μg/mL streptomycin. The results were measured through the Sulforhodamine B method, where the amount of pigmented cells is directly proportional to the total protein mass and therefore to the number of bounded cells. Results were expressed as GI_50_ values (concentration that inhibits 50% of cell growth) and Ellipticine was used as a positive control.

#### 2.3.2. Cytotoxicity in Human Tumor Cell Lines

Four human tumor cell lines were used: MCF-7 (breast adenocarcinoma), NCI-H460 (non-small cell lung cancer), HeLa (cervical carcinoma) and HepG2 (hepatocellular carcinoma), as previously described by Barros et al. [23]. MCF-7, HeLa and HepG2 were obtained from the European Collection of Cell Cultures (ECACC, Salisbury, UK) and NCI-H460 was kindly provided by the National Cancer Institute (NCI, Bethesda, MD, USA). Extracts were tested at a final concentration range from 400 to 1.56 μg/mL. RPMI-1640 medium containing 10% heat-inactivated FBS and 2 mM glutamine was used to routinely maintain the adherent cell cultures at 37 °C, in a humidified air incubator containing 5% CO_2_. For the experiments, each cell line was placed at an appropriate density (1.0 × 10^4^ cells/well) into 96-well plates. The cell growth inhibition was measured using sulforhodamine B, results were expressed as GI_50_ values and Ellipticine was used as a positive control.

### 2.4. Statistical Analysis

The experimental design was a completely randomized design (CRD), with three replications per treatment (harvesting stage, plant part). For the statistical analysis of chemical composition and bioactivity assays, three samples were analyzed for each treatment, whereas all of the assays were carried out in triplicate. Statistical analysis was conducted with the aid of Statgraphics 5.1.plus (Statpoint Technologies, Inc., VA, USA). Data were evaluated by a two-way ANOVA for the main effects, whereas the means of values were compared by Tukey’s honestly significant difference (HSD) test (*p* = 0.05) in the case of harvesting stage effect, and by Student’s *t*-test (*p* = 0.05) in the case of plant part effect. 

## 3. Results and Discussion

The statistical analysis of the data showed a significant interaction between plant parts and harvesting stage for all the recorded parameters. Therefore, the interpretation of the data refers to the combined effect of both factors. Moisture content and proximate analysis results are presented in Table 1. The moisture content of leaves was the highest at 29 and 43 DAS and decreased at 52 DAS with plant maturity, whereas stems contained more water at 43 DAS, followed by the harvesting stages of 52 and 29 DAS. For macronutrient content (fat, proteins, ash, and carbohydrates) and the energetic value of plant tissues, the highest content in leaves was observed at the last harvest (52 DAS), which could be probably attributed to a concentration effect. Moreover, the opposite trend was observed in the case of stems, where harvesting at 29 DAS resulted in the highest content of ash and carbohydrates and the highest energetic value. However, fat content did not differ significantly between the first (29 DAS) and the last harvest (52 DAS), whereas protein content was the highest at the last harvest (52 DAS). Similar trends regarding the macronutrient content at different harvesting stages have been previously reported by Mohamed and Hussein [5], who also suggested an increase in fat, protein and ash content with increasing maturity, whereas they also reported an opposite trend for carbohydrate content. The values for moisture and macronutrient content detected in our study were in the same range as those reported by Ezekwe et al. [17], who studied eight purslane accessions planted at different dates, as well with the study described by Teixeira and Carvalho [13], who evaluated the effect of salinity on the proximate composition of *Portulaca oleracea* cv. “Golden leaf” for two growing seasons. The only difference with the above-mentioned reports was observed in lipid content, which was lower in our study. This finding could be attributed to differences in genotype, harvesting stage and growth conditions. According to Jin et al. [10], stress conditions such as heat, drought and salinity may accelerate protein catabolism and result in lower protein content. In addition, Teixeira and Carvalho [13] suggested that apart from harvesting stage (49 and 57 DAS), growing season (spring and summer) may also have an effect on the proximate composition of purslane leaves.

Significant differences in moisture content were also observed between both plant parts, with leaves having a higher moisture content only at the first harvest stage (29 DAS), since developing plants gradually become succulent and contain more water in stems than leaves (Table 1). Similar results have been reported by Oliveira et al. [4], who also detected higher moisture content in leaves compared to stems, regardless of purslane genotype. Macronutrient content and energetic value also differed between plant parts at the studied harvesting stages. In particular, leaves contained more fat and proteins at the first harvest, whereas stems had a higher content of carbohydrates and ash and a higher energetic value at the same harvesting stage. Moreover, leaves had higher macronutrient content and energetic value than stems at the second and the last harvesting stages (apart from carbohydrate content at the second harvest), which could be partly attributed to the lower moisture content values and the resulting concentration effect. Oliveira et al. [4] also reported differences in fat content between leaves and stems of purslane plants from four different locations in Northern Portugal, whereas there were significant differences in the reported values compared to our study, probably because plants were collected in the wild instead of being cultivated. Moreover, no details regarding the harvesting stage were available in the study of Oliveira et al. [4] in order to make direct comparisons with our study. According to Ezeabara et al. [27], moisture, fat, protein and ash contents were higher in leaves than in stems, when plants were harvested at flowering stage, whereas the opposite trend was observed for carbohydrate content. These results are in agreement with the values of the last harvest of our study, indicating that late harvests increase the nutritional value of leaves in comparison to stems, due to maturation progress and the decrease in moisture content. 

The contents of individual and total tocopherols in relation to the harvesting stage and plant part are presented in Table 2. Tocopherols, also known as vitamin E, are significant bioactive compounds with antioxidant properties against lipid peroxidation of biological membranes [28]. α-tocopherol was the main detected isoform in both leaves and stems, followed by γ-, β- and δ-tocopherols. Leaves contained significantly higher amounts of all isoforms and total tocopherols, regardless of the harvesting stage, while harvesting at later stages (52 DAS) had a beneficial effect on the content of most of the individual and total tocopherols. The only exception was observed for γ-tocopherol content, which was the highest at early growth stages (29 DAS). A contrasting trend was observed in the case of stems, where the highest content of individual and total tocopherols was recorded at the stage of 29 DAS, except for β-tocopherol where no significant differences were observed between harvesting at 29 and 43 DAS. Similarly to our study, Szalai et al. [7] reported that α- and γ-tocopherols were the main detected tocopherols in fully mature leaves of three purslane microspecies, although they did not detect the other two vitamin E isoforms (β- and δ-tocopherols) that were present in our study. Moreover, the highest content of α- and total tocopherols at the last harvesting stage (52 DAS) could be attributed to unfavorable growing conditions (namely, high temperatures and high transpiration), which could induce tocopherols’ biosynthesis as a means of plants’ antioxidant defense [29]. 

The free sugar composition of leaves and stems in relation to harvesting stage is presented in Table 2. The main detected sugars were glucose and fructose, followed by sucrose and trehalose, which were detected in lower amounts. Stems contained significantly higher amounts of fructose, glucose, sucrose and total free sugars than leaves, regardless of harvesting stage, whereas trehalose content was higher in leaves compared to stems when harvesting took place at late growth stages (43 and 52 DAS). In addition, an increase in glucose and fructose content was observed at late growth stages (43 and 52 DAS) for both leaves and stems, which was also depicted in the total sugar content of plant parts. Sugar composition was similar to our previously reported study, where six different purslane genotypes were evaluated in terms of chemical composition [15], whereas any differences in individual sugars’ content could be explained mostly by genotypic differences and barely by the environmental conditions and cultivation practices, which were identical in both studies. Moreover, Mohamed and Hussein [5] suggested glucose as the main detected sugar, while fluctuating trends were observed in sugar content between plant parts at different growth stages (30, 49 and 59 days after planting). The lower amounts of sucrose in leaves compared to fructose and glucose could be attributed to the use of fixed carbon for the biosynthesis of fructose and glucose and the concurrent export of sucrose from leaves to be used as a biosynthetic substrate [30]. 

The organic acid content of plant parts in relation to harvesting stage is presented in Table 3. Four organic acids were detected in leaves and stems, namely oxalic, malic, quinic and citric acids, regardless of the harvesting stage. However, the composition of individual organic acids differed significantly between plant parts and harvesting stages. In particular, leaves contained mostly quinic and oxalic acids at all the harvesting stages, although quinic acid content was significantly higher at the last growth stage (52 DAS). On the other hand, stems contained oxalic, quinic and malic acids at the earliest growth stage (29 DAS), whereas quinic acid decreased significantly with plant maturity. Similarly, Oliveira et al. [4], who studied organic acid content in the leaves and stems of different purslane genotypes, detected significant differences between plant parts, although the magnitude of these differences varied depending on the genotype. In contrast to our study, Szalai et al. [7] detected oxalic, malic and ascorbic acids in the leaves of three purslane microspecies, a difference that could be attributed mostly to the effect of harvesting stage. However, it is worth mentioning the effect of genotype on organic acid profile and content, which is also important and has been already confirmed in previous studies [4,15]. 

Purslane is considered as one of the richest plant sources of omega-3 fatty acids. The composition of leaves and stems in relation to harvesting stage is presented in Table 4. The main detected fatty acids in both stems and leaves were linoleic, palmitic, α-linolenic, behenic, oleic and lignoceric acids, although the abundance of individual compounds varied among the studied plant parts and harvesting stages. The major compound in stems was palmitic acid, followed by linoleic and α-linolenic acids. In addition, the highest content of palmitic and linoleic acids was detected at 29 DAS, whereas α-linolenic acid content was the highest at 43 DAS. In contrast, the fatty acid profile of leaves differed significantly from that of stems, with α-linolenic acid contributing the most to the overall fatty acid content, especially at the first two harvesting stages (29 and 43 DAS). The same fatty acids have been previously reported by Oliveira et al. [4], who also suggested α-linolenic, palmitic, oleic, stearic and behenic acids as the main fatty acids in leaves. However, the reported profile of individual fatty acids differed from that in our study, without additional information regarding harvesting stage being available. Similarly, Guil-Guerrero and Rodríguez-García [31] suggested a different profile of fatty acids in phospholipid and neutral lipid fractions of purslane leaves, which contained higher amounts of omega-6 (n6) than omega-3 (n3) fatty acids. Moreover, in our study the overall α-linolenic acid content (the sum of the contents of stems and leaves) was the highest (0.1 g/100 g fw) at the earliest harvesting stage (29 DAS), despite the significantly higher fat content of leaves at the late harvesting stage (0.1 g/100 g fw at 52 DAS; see Table 1). Similar amounts of α-linolenic acid (1.06 g/100 g dw) were reported by Guil-Guerrero and Rodríguez-García [31], although they also suggested that purslane leaves contain higher amounts of linoleic than α-linolenic acid. The highest polyunsaturated fatty acids (PUFA)/saturated fatty acids (SFA) ratio was observed in stems and leaves at the middle (43 DAS) and late harvesting stages (52 DAS), respectively. In contrast, the lowest n6/n3 fatty acid ratio in stems and leaves was recorded at the earliest harvesting stage (29 DAS). In addition, for all the studied harvesting stages and plant parts, the PUFA/SFA ratio was higher than 0.45, while the n6/n3 ratio was lower than 4.0, indicating a high nutritional value of the edible plant parts [32]. Moreover, the detected values for the above-mentioned ratios of our study were within the same range as the ones recorded in the study of Fontana et al. [19]. 

**Figure 1:**
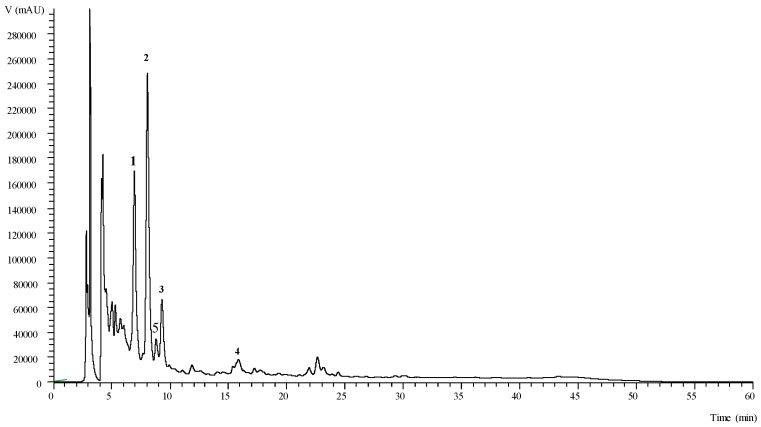
Chromatographic profile of the hydroethanolic extract obtained from purslane stems, recorded at 330 nm. Peak numbers correspond to the compounds already mentioned in Table 5.

The identified phenolic compounds and oleracein derivatives in both plant parts of purslane are presented in Table 5, while quantification data are presented in Table 6 and Figure 1. A total of five compounds were identified in the hydroethanolic extracts of purslane aerial plant parts (Table 5). These compounds included three phenolic acids (caffeic acid derivatives, peaks 2, 3 and 4) and two oleracein derivatives (phenolic alkaloids, peaks 1 and 5). Caffeic acid (peak 2) was identified in comparison with a commercial standard, whereas compound 4 was identified as a caffeic acid derivative, thus its pseudomolecular ion was not clearly identified due to the very small amounts present. Compound 3 ([M − H]^−^ at *m/z* 385) presented a unique MS² fragment at *m/z* 223 (sinapic acid), with a loss of a hexosyl moiety (162 u), being tentatively identified as sinapic acid hexoside. Compounds 1 ([M − H]^−^ at *m/z* 664) and 5 ([M − H]^−^ at *m/z* 502) corresponded to oleracein derivatives, being identified as oleracein C and A, respectively, taking into account previous findings in the literature regarding *P. oleracea* [33,34]. Oleraceins are cyclodopa alkaloids, which have been previously reported by Xiang et al. [35], who identified five oleraceins in dried purslane plants, among other compounds. Moreover, Farag and Shakour [36] suggested that the presence of these compounds in purslane aerial parts may be used as a criterion for the classification of different *Portulaca* taxa. To the best of our knowledge, the rest of the identified compounds have not been previously reported in purslane aerial parts. 

The composition of phenolic compounds and oleracein derivatives differed among the tested plant parts and harvesting stages (Table 6). Concerning the quantification of oleraceins, *p*-coumaric acid was the standard applied to perform the quantitative analysis (an available compound with a similar structure); therefore, these compounds were quantified as equivalents to this phenolic acid. Leaves contained significantly higher amounts of individual and total phenolic compounds and oleracein derivatives compared to stems, regardless of harvesting stage. Moreover, harvesting at early stages (29 DAS) resulted in significantly higher contents of phenolic compounds and oleracein derivatives, especially in the case of leaves. Meanwhile, in stems the content of oleracein C was the highest at the same harvesting stage (29 DAS), resulting in the highest total contents of phenolic compounds and oleracein derivatives, accordingly. As mentioned previously for the case of tocopherols (see Table 2), phenolic compounds and oleracein derivatives may also contribute to the overall defense mechanisms of purslane; therefore, the increased content at the earlier stages could be attributed to the protective purposes of developing leaves. Similar results regarding the effect of harvesting stage on the compositions of phenolic compounds and oleracein derivatives have been reported by Lim and Quah [37], who also observed a decrease in total phenolic compounds and oleracein derivatives in leaves with increasing maturity. The differences in the compositions of phenolic compounds and oleracein derivatives could be attributed to differences in the tested genotypes [35,37], to different cultivation regimes and growing conditions [14,18] and also to extraction protocols [38]. 

The cytotoxic effects on PLP2 non-tumor cell lines of the samples showed no hepatotoxicity, with GI_50_ values being higher than 400 μg/mL for all the harvesting stages and plant parts (data not shown). Concerning the evaluation of in vitro activity against the four tumor cells lines, the extracts did not present any activity at the tested concentrations (data not show), with GI_50_ values being higher than 400 μg/mL.

The hepatoprotective effects of ethanolic extracts of purslane aerial parts have been previously reported by Eidi et al. [39], whose findings are in agreement with the results of our study. Similarly, ethanolic and aqueous extracts of air-dried purslane leaves showed hepatoprotective properties against paracetamol-induced liver damage [40]. Moreover, previous studies have suggested weak to moderate inhibitory effects against two mutagens (benzo[a]pyrene (B[a]P) and 2-Amino-3-methyl-imidazo[4,5-f]quinolone (IQ)) and no mutagenic activity on Salmonella typhimurium [41], while Choi and Ryeom [42] and Zhao et al. [43] reported significant antitumor activity against leukaemia and cervical carcinoma cell lines, respectively.

## 4. Conclusions

The results of the present study showed a significant effect of plant parts and harvesting stages on the nutritional value and chemical composition of purslane. Leaves contained higher amounts of macronutrients than stems, especially at 52 DAS, while α-tocopherol was the main vitamin E isoform, which increased at 52 DAS resulting in the highest overall tocopherol content. Glucose and fructose were the main sugars in stems and leaves, respectively, while stems contained higher amounts of total sugars in comparison to leaves. Regarding oxalic acid as well as total organic acids, the highest contents were recorded in leaves, especially at the last harvesting stage (52 DAS). The edible plant parts contained both omega-6 and omega-3 fatty acids, although leaves were more abundant in α-linolenic acid than stems, which contained mostly palmitic and linoleic acids. Phenolic compounds and oleracein derivatives were also detected in plant parts, with oleraceins A and C being the main compounds detected in leaves, regardless of harvesting stage. In conclusion, early harvesting and the separation of plant parts could increase the nutritional value of the final product through increasing the content of valuable compounds, such as omega-3 fatty acids, phenolic compounds and oleracein derivatives, while at the same time, the contents of anti-nutritional compounds such as oxalic acid are reduced.

## Figures and Tables

**Table 1:** Nutritional value (g/100 g fresh weight (fw)) and energetic value (kcal/100 g fw) of purslane stems and leaves in relation to harvesting stage (mean ± SD).

Harvest Stage (DAS) *	Plant Part	Moisture (%)	Fat	Proteins	Ash	Carbohydrates	Energy
29	Stems	88.49 ± 0.41c	0.111 ± 0.002a	1.31 ± 0.01b	2.48 ± 0.09a	7.6 ± 0.1a	47 ± 9a
	Leaves	91.00 ± 0.49a	0.157 ± 0.001b	1.57 ± 0.02c	2.14 ± 0.05b	5.13 ± 0.02c	43.2 ± 0.1c
43	Stems	91.97 ± 0.08a	0.091 ± 0.001b	0.76 ± 0.01c	1.78 ± 0.03b	5.40 ± 0.02b	35.54 ± 0.03c
	Leaves	90.81 ± 0.16a	0.148 ± 0.002b	1.91 ± 0.01b	1.89 ± 0.05c	5.25 ± 0.03b	45.70 ± 0.02b
52	Stems	91.31 ± 0.1b	0.111 ± 0.003a	1.44 ± 0.01a	1.74 ± 0.04b	5.39 ± 0.03b	41.52 ± 0.02b
	Leaves	88.16 ± 0.41b	0.230 ± 0.001a	2.96 ± 0.04a	2.40 ± 0.06a	6.2 ± 0.1a	61.3 ± 0.1a
Student’s *t*	Plant Part **	<0.001	<0.001	<0.01	<0.01	<0.01	<0.01

* DAS: days after sowing. Different Latin letters (a–c) in the same column refer to significant differences between harvest stages for the same plant part (stems or leaves) at *p* = 0.05. ** Comparison of means of different plant parts (stems and leaves) from the same harvest was performed with Student’s *t*-test at *p* = 0.05.

**Table 2:** Composition in tocopherols (µg/100 g fw) and sugars (g/100 g fw) of purslane stems and leaves in relation to harvesting stage (mean ± SD).

**Harvest Stage** (DAS) *	**Plant Part**	**α-Tocopherol**	**β-Tocopherol**	**γ-Tocopherol**	**δ-Tocopherol**	**Total Tocopherols**
29	Stems	26.0 ± 0.2a	3.4 ± 0.3a	14.4 ± 0.1a	0.99 ± 0.03	44.7 ± 0.5a
	Leaves	215 ± 4b	14.0 ± 0.7b	140.7 ± 0.1a	9.6 ± 0.5b	380 ± 4b
43	Stems	19.3 ± 0.5b	3.6 ± 0.6a	8.3 ± 0.1b	nd	31 ± 1b
	Leaves	197 ± 3c	12.4 ± 0.2b	87.7 ± 0.2c	5.1 ± 0.2c	302 ± 2c
52	Stems	10.4 ± 0.2c	1.8 ± 0.1b	5.2 ± 0.2c	nd	17.4 ± 0.6c
	Leaves	327 ± 3a	44 ± 2a	97 ± 8b	13.5 ± 0.5a	481 ± 9a
Student’s *t*	Plant part	<0.001	<0.001	<0.001	<0.001	<0.001
**Harvest Stage** (DAS) *	**Plant Part**	**Fructose**	**Glucose**	**Sucrose**	**Trehalose**	**Total Sugars**
29	Stems	0.308 ± 0.006c	0.358 ± 0.001c	0.135 ± 0.001a	0.024 ± 0.001a	0.830 ± 0.006c
	Leaves	0.11 ± 0.01b	0.041 ± 0.002c	nd	0.012 ± 0.001c	0.160 ± 0.007b
43	Stems	0.44 ± 0.02a	0.53 ± 0.02b	0.051 ± 0.001c	0.012 ± 0.001b	1.03 ± 0.04b
	Leaves	0.183 ± 0.007a	0.113 ± 0.002a	0.009 ± 0.001a	0.026 ± 0.001b	0.330 ± 0.009a
52	Stems	0.39 ± 0.01b	0.74 ± 0.02a	0.118 ± 0.003b	0.022 ± 0.001a	1.28 ± 0.04a
	Leaves	0.179 ± 0.007a	0.100 ± 0.001b	0.014 ± 0.001a	0.041 ± 0.001a	0.330 ± 0.008a
Student’s *t*	Plant part **	<0.001	<0.001	<0.001	<0.001	<0.001

nd: not detected; * DAS: days after sowing; Different Latin letters (a–c) in the same column refer to significant differences between harvest stages for the same plant part (stems or leaves) at *p* = 0.05. ** Comparison of means of different plant parts (stems and leaves) from the same harvest was performed with Student’s *t*-test at *p* = 0.05.

**Table 3:** Composition in organic acids (g/100 g fw) of purslane stems and leaves in relation to harvesting stage (mean ± SD).

Harvest Stage (DAS) *	Plant Part	Oxalic Acid	Quinic Acid	Malic Acid	Citric Acid	Total Organic Acids
29	Stems	7.70 ± 0.07a	6.36 ± 0.04a	6.39 ± 0.07b	5.00 ± 0.03a	25.5 ± 0.2a
	Leaves	6.2 ± 0.1b	6.82 ± 0.01c	3.00 ± 0.03a	3.26 ± 0.01a	19.2 ± 0.1b
43	Stems	4.77 ± 0.01c	1.31 ± 0.01c	6.56 ± 0.07a	1.57 ± 0.04c ^¥^	14.22 ± 0.04c
	Leaves	5.7 ± 0.1c	8.4 ± 0.2b	1.90 ± 0.04b	1.53 ± 0.02b ^¥^	17.6 ± 0.1c
52	Stems	7.16 ± 0.02b	3.57 ± 0.04b	5.38 ± 0.06c	2.78 ± 0.01b	18.89 ± 0.04b
	Leaves	8.6 ± 0.2a	16.8 ± 0.5a	1.67 ± 0.01c	3.24 ± 0.03a	30.3 ± 0.2a
80	Seeds	0.470 ± 0.005	nd	tr	tr	0.470 ± 0.005
Student’s *t*	Plant part **	<0.001	<0.001	<0.001	<0.001	<0.001

nd: not detected; tr: traces; * DAS: days after sowing; ^¥^: no significant difference was observed between plant parts. Different Latin letters (a–c) in the same column refer to significant differences between harvest stages for the same plant part (stems or leaves) at *p* = 0.05. ** Comparison of means of different plant parts (stems and leaves) from the same harvest was performed with Student’s *t*-test at *p* = 0.05.

**Table 4:** Fatty acid composition (%) of the studied purslane stems and leaves (mean ± SD) in relation to harvesting stage.

Fatty Acids	29 DAS		43 DAS		52 DAS		Student’s *t*
	Stems	Leaves	Stems	Leaves	Stems	Leaves	Plant Parts **
C6:0	0.543 ± 0.001a	0.024 ± 0.001c	0.30 ± 0.03b	0.067 ± 0.001b	0.280 ± 0.006c	0.220 ± 0.001a	<0.001
C8:0	0.098 ± 0.003c	0.032 ± 0.003c	0.12 ± 0.01a	0.039 ± 0.001b	0.115 ± 0.008b	0.095 ± 0.007a	<0.001
C10:0	0.100 ± 0.003a	0.052 ± 0.001b	0.091 ± 0.004b	0.051 ± 0.001b	0.094 ± 0.006b	0.125 ± 0.007a	<0.001
C12:0	0.93 ± 0.03a	0.81 ± 0.02c	0.62 ± 0.04b	0.867 ± 0.001b	0.640 ± 0.006b	1.37 ± 0.04a	<0.001
C14:0	1.42 ± 0.02a	0.736 ± 0.002c	0.97 ± 0.02b	0.77 ± 0.01b	0.85 ± 0.02c	1.24 ± 0.01a	<0.001
C15:0	0.45 ± 0.01b	0.49 ± 0.01b	0.472 ± 0.008b	0.420 ± 0.003c	0.62 ± 0.02a	0.75 ± 0.01a	<0.001
C16:0	21.8 ± 0.2a	9.8 ± 0.1c	21.0 ± 0.1b	10.83 ± 0.01b	20.2 ± 0.2c	12.39 ± 0.03a	<0.001
C16:1	0.236 ± 0.009c	0.52 ± 0.01b	0.36 ± 0.01b	0.48 ± 0.01c	0.401 ± 0.009a	0.730 ± 0.001a	<0.001
C17:0	0.54 ± 0.03b	0.15 ± 0.01c	0.48 ± 0.02c	0.159 ± 0.005b	0.816 ± 0.007a	0.265 ± 0.007a	<0.001
C18:0	4.9 ± 0.1a	2.52 ± 0.05c	4.55 ± 0.05c	2.72 ± 0.01b	4.83 ± 0.07b	3.89 ± 0.06a	<0.001
C18:1n9c+t	9.55 ± 0.04b	5.29 ± 0.05b	11.62 ± 0.01a	4.65 ± 0.04c	6.83 ± 0.14c	6.4 ± 0.1a	<0.001
C18:2n6c	23.02 ± 0.02c	11.40 ± 0.08c	27.11 ± 0.02a	11.63 ± 0.02b	25.4 ± 0.2b	14.81 ± 0.02a	<0.001
C18:3n3	17.31 ± 0.04a	54.92 ± 0.08a	15.03 ± 0.07b	54.34 ± 0.03a	11.64 ± 0.01c	35.4 ± 0.1b	<0.001
C20:0	1.86 ± 0.06c	1.79 ± 0.01b	1.99 ± 0.03c	1.80 ± 0.01b	2.2 ± 0.1a	2.95 ± 0.03a	<0.001
C20:1CIS-11	0.40 ± 0.01a	0.08 ± 0.01c	0.258 ± 0.006b	0.11 ± 0.01b	0.146 ± 0.001c ^¥^	0.140 ± 0.001a ^¥^	<0.001
C20:3n3+C21:0	0.67 ± 0.01a	0.155 ± 0.004c	0.63 ± 0.04c	0.195 ± 0.004b	0.646 ± 0.002b	0.32 ± 0.02a	<0.001
C20:5n3	0.17 ± 0.01a	0.051 ± 0.003a	0.17 ± 0.01a	0.042 ± 0.001b	0.146 ± 0.003b	0.040 ± 0.001b	<0.001
C22:0	11.0 ± 0.2b	9.0 ± 0.3b	9.6 ± 0.2c	8.62 ± 0.09c	15.40 ± 0.23a	15.0 ± 0.2a	<0.001
C23:0	0.44 ± 0.02c	0.20 ± 0.01b	0.57 ± 0.01b	0.15 ± 0.01c	0.77 ± 0.02a	0.31 ± 0.01a	<0.001
C24:0	4.5 ± 0.1b	2.04 ± 0.08b	4.1 ± 0.2c	2.05 ± 0.01b	7.97 ± 0.02a	3.61 ± 0.04a	<0.001
Total SFA (% of total FA)	48.65 ± 0.02b	27.58 ± 0.06c	44.82 ± 0.01c	28.5 ± 0.1b	54.8 ± 0.1a	42.2 ± 0.3a	<0.001
Total MUFA (% of total FA)	10.18 ± 0.04b	5.89 ± 0.05b	12.24 ± 0.02a	5.25 ± 0.06c	7.38 ± 0.13c ^¥^	7.3 ± 0.1a ^¥^	<0.001
Total PUFA (% of total FA)	41.17 ± 0.02b	66.53 ± 0.01a	42.94 ± 0.02a	66.21 ± 0.04b	37.81 ± 0.25c	50.5 ± 0.2c	<0.001
PUFA/SFA	0.864 ± 0.001b	2.412 ± 0.003a	0.958 ± 0.001a	2.319 ± 0.007b	0.690 ± 0.004c	1.196 ± 0.009c	<0.001
n6/n3	1.269 ± 0.005c	0.207 ± 0.001c	1.71 ± 0.01b	0.213 ± 0.001b	2.04 ± 0.01a	0.414 ± 0.002a	<0.001

* DAS: days after sowing; ^¥^: no significant difference was observed between plant parts. Caproic acid (C6:0); Caprylic acid (C8:0); Capric acid (C10:0); Lauric acid (C12:0); Myristic acid (C14:0); Pentadecylic acid (C15:0); Palmitic acid (C16:0); Palmitoleic acid (C16:1); Margaric acid (C17:0); Stearic acid (C18:0); Oleic acid (C18:1n9); Linoleic acid (C18:2n6c); α-Linolenic acid (C18:3n3); Arachidic acid (C20:0); Eicosenoic acid (C20:1CIS-11); Eicosatrienoic acid (C20:3n3); Heneicosylic acid (C21:0); Eicosapentaeonic acid (C20:5n3); Behenic acid (C22:0); Tricosylic acid (C23:0); Lignoceric acid (C24:0); SFA: saturated fatty acids; MUFA: monounsaturated fatty acids; PUFA: polyunsaturated fatty acids; n6/n3: omega-6/omega-3 fatty acids. Different Latin letters (a–c) in the same row refer to significant differences between harvest stages for the same plant part (stems or leaves) at *p* = 0.05. ** Comparison of means of different plant parts (stems and leaves) from the same harvest was performed with Student’s *t*-test at *p* = 0.05.

**Table 5:** Retention time (Rt), wavelengths of maximum absorption in the UV-VIS region (λ_max_), mass spectral data, identification and quantification of phenolic compounds and oleracein derivatives in purslane aerial plant parts (leaves and stems).

Peak	Rt (min)	λ_max_ (nm)	[M−H]^−^ (*m/z*)	MS^2^ (*m/z*)	Tentative Identification
1	6.82	345	664	502(100), 340(20), 296(5), 194(3)	Oleracein C
2	8.77	323	179	161(100), 143(63), 119(40)	Caffeic acid
3	9.29	329	385	223 (100)	Sinapic acid hexoside
4	11.89	318	-	179(100), 161(55), 143(31), 119(18)	Caffeic acid derivative
5	15.54	338	502	340(100), 296(5), 194(3), 145(3)	Oleracein A

**Table 6:** Quantification of phenolic compounds and oleracein derivatives in purslane stems and leaves (mg/100 g dried weight (dw)) in relation to harvesting stage (mean ± SD).

Peak	Phenolic Compound	29		43		52		Plant Parts **
		Stems	Leaves	Stems	Leaves	Stems	Leaves	
1	Oleracein C ^A^	15.2 ± 0.5a	143 ± 5a	6.7 ± 0.1b	21.2 ± 0.3c	3.34 ± 0.07c	102 ± 2b	<0.01
2	Caffeic acid ^B^	0.44 ± 0.02a	nd	0.45 ± 0.01a	nd	tr	nd	<0.01
3	Sinapic acid hexoside ^C^	4.2 ± 0.1a	22.1 ± 0.7a	4.3 ± 0.2a	nd	1.37 ± 0.03b	nd	<0.01
4	Cafferic acid derivative	nd	nd	nd	nd	tr	nd	-
5	Oleracein A ^A^	nd	103 ± 2a	0.75 ± 0.01a	8.2 ± 0.1c	0.28 ± 0.02b	34.9 ± 0.8b	<0.01
	TPCOD	19.8 ± 0.4a	268 ± 6a	12.2 ± 0.1b	29.3 ± 0.4c	4.99 ± 0.08c	137 ± 3b	<0.01

* DAS: days after sowing; nd: not detected; TPCOD: Total phenolic compounds and oleracein derivatives; tr: traces. Calibration curves used: ^A^: *p*-coumaric acid (*y* = 301,950*x* + 6966.7; *R²* = 0.999); ^B^: caffeic acid (*y* = 388,345*x* + 406,369; *R²* = 0.999); ^C^: sinapic acid (*y* = 197,337*x* + 30,036; *R²* = 0.999). Different Latin letters (a–c) in the same column refer to significant differences between harvest stages for the same plant part (stems and leaves) at *p =* 0.05. ** Comparison of means of different plant parts (stems and leaves) from the same harvest was performed with Student’s *t*-test at *p* = 0.05.
